# A Randomized Trial to Improve Adherence to Follow-up Eye Examinations Among People With Glaucoma

**DOI:** 10.5888/pcd18.200567

**Published:** 2021-05-20

**Authors:** Benjamin E. Leiby, Sarah E. Hegarty, Tingting Zhan, Jonathan S. Myers, L. Jay Katz, Julia A. Haller, Michael Waisbourd, Christine Burns, Meskerem Divers, Jeanne Molineaux, Jeffrey Henderer, Charles Brodowski, Lisa A. Hark

**Affiliations:** 1 Thomas Jefferson University, Sidney Kimmel Medical College, Department of Pharmacology and Experimental Therapeutics, Division of Biostatistics, Philadelphia, Pennsylvania; 2 Wills Eye Hospital, Glaucoma Research Center, Philadelphia, Pennsylvania; 3 Thomas Jefferson University, Sidney Kimmel Medical College, Department of Ophthalmology, Philadelphia, Pennsylvania; 4 Wills Eye Hospital, Office of the Ophthalmologist-in-Chief, Philadelphia, Pennsylvania; 5 Department of Ophthalmology, Tel Aviv Medical Center, affiliated with the Sackler Faculty of Medicine, Tel Aviv University, Israel; 6 Temple University School of Medicine, Department of Ophthalmology, Philadelphia, Pennsylvania; 7 Columbia University Irving Medical Center, Department of Ophthalmology, New York, New York

## Abstract

**Introduction:**

Appointment nonadherence is common among people with glaucoma, making it difficult for eye care providers to monitor glaucoma progression. Our objective was to determine whether the use of patient navigators, in conjunction with social worker support, could increase adherence to recommended follow-up eye appointments.

**Methods:**

A randomized, controlled trial evaluated the effectiveness of an intervention that used patient navigators and social workers to improve patient adherence to follow-up eye care compared with usual care. Participants with glaucoma and other eye diseases (N = 344) were identified at primary care clinics in community settings through telemedicine screening of imaging and then randomized to enhanced intervention (EI) or usual care (UC). Data on participants’ visits with local ophthalmologists were collected for up to 3 years from randomization. Groups were compared for timely attendance at the first visit with the local ophthalmologist and adherence to recommended follow-up visits.

**Results:**

Timely attendance at the first visit was higher for EI than UC (74.4% vs 39.0%; average relative risk [aRR] = 1.85; 95% CI, 1.51–2.28; *P* < .001). Rates of adherence to recommended annual follow-up during year 1 were 18.6% in the EI group and 8.1% in the usual care group (aRR = 2.08; 95% CI, 1.14–3.76; *P* = .02). The aRR across years 2 and 3 was 3.92 (95% CI, 1.24–12.43; *P* = .02).

**Conclusion:**

An intervention using patient navigators and social workers doubled the rate of adherence to annual recommended follow-up eye care compared with usual care in community settings, and was effective at increasing connections with local ophthalmologists. Interventions to further improve long-term adherence are needed.

**Summary**

**What is already known on this topic?**

Nonadherence to follow-up eye care is common among people with glaucoma and other eye diseases. Use of patient navigators and social workers can increase adherence to eye care appointments.

**What is added by this report?**

The results of a randomized, controlled trial of patients with glaucoma and other eye diseases showed that a patient navigation and social work intervention doubled the rate of follow-up adherence in community settings.

**What are the implications for public health practice?**

Involving patient navigators and social workers in ophthalmic care could improve care and reduce disease progression.

## Introduction

Glaucoma is a chronic eye disease resulting in visual field defects and progressive vision loss and is the leading cause of irreversible blindness worldwide (1). Among other chronic diseases, diabetes in particular is associated with increased likelihood of developing glaucoma (2,3). Because glaucoma is asymptomatic in early stages, early detection and timely intervention are critical to prevent vision loss (4). Nonadherence to recommended follow-up eye examinations reduces care and worsens outcomes (5). Fifty-four percent of people diagnosed with glaucoma fail to attend follow-up eye-related appointments (6). Barriers to nonadherence include health care costs, lack of transportation, and emotional distress (7,8). These barriers most often affect people of color, who have lower attendance rates at follow-up eye care appointments than White patients (9–11).

Patient navigators and social workers can help address barriers to appointment adherence. Patient navigators direct patients to appropriate health care resources, coordinate and schedule appointments, verify insurance status, and arrange transportation (12,13). Patient navigation programs have been used in other medical fields to promote adherence to medication and treatment (14–16). Few studies have looked at using patient navigators to improve appointment adherence among glaucoma patients, particularly among previously undiagnosed people with risk factors for glaucoma and eye disease (12,17).

Social workers assess, track, and lessen psychosocial barriers to care to improve quality of life and patient well-being (7,18). Social workers not only help patients navigate the health care system; they also provide emotional support, which has been shown to increase appointment adherence (18–20). In one study, a medical social worker in a pediatric ophthalmology setting increased appointment adherence by 45% (19,20). In several observational studies, glaucoma patients reported that a social worker resolved their issues and supported their keeping appointments with their ophthalmologist (7,21).

The combined use of social workers and patient navigators to improve appointment adherence among glaucoma patients has not been investigated previously in a controlled, prospective adult study. Our objective was to determine whether the use of patient navigators and social workers could increase adherence to recommended follow-up eye appointments among a high-risk population with glaucoma or other eye diseases.

## Methods

### Study design

The Philadelphia Telemedicine Glaucoma Detection and Follow-up Study was a prospective, randomized clinical trial that aimed to address the issue of poor adherence to follow-up eye examinations by providing patient navigator and social worker support to directly guide participants through the eye care process (22). The 5-year study was conducted by Wills Eye Hospital, funded by the Centers for Disease Control and Prevention, and registered with ClinicalTrials.gov (NCT02390245). As described previously (22), the study’s 2 phases 1) conducted a practice-based telemedicine screening program for glaucoma and other eye diseases among underserved populations with risk factors for eye disease and 2) evaluated whether a community intervention with patient navigation and social worker support improved access to and use of eye care. The study was approved by the Wills Eye Hospital Institutional Review Board and was conducted in accordance with the Declaration of Helsinki. Written informed consent was obtained from all participants before each phase of the study.

In Phase 1, the study aimed to identify people with undiagnosed glaucoma and other eye diseases and facilitate their referral to local ophthalmologists. A targeted sample at high risk for eye disease was recruited from 12 community partner organizations and consisted of African American, Hispanic, and Asian adults over age 40; adults over age 65 of any race/ethnicity; and people over age 40 with a family history of glaucoma or currently diagnosed with diabetes. We enrolled only people who had not seen an ophthalmologist in the previous 12 months (N = 906). After informed consent was obtained, participants underwent a brief vision screening in their primary care provider’s (PCP’s) office (Visit 1), which included measuring visual acuity and intraocular pressure (IOP), and using fundus (retina) photography. Both retina and glaucoma specialists used telemedicine to read the images at Wills Eye Hospital. If the IOP was greater than 30 mm Hg, participants were immediately referred to a local ophthalmologist (fast-tracked). Otherwise, participants with findings suggestive of sight-threatening disease, such as glaucoma, diabetic retinopathy, or hypertensive complications, or with unclear screening results were invited to return to the same location for a comprehensive eye examination by an ophthalmologist (Visit 2). At Visit 2, visual acuity and IOP were assessed again in addition to an ophthalmologic examination. Visual field tests were also performed, and vision-related quality of life was assessed by using the National Eye Institute Vision Function Questionnaire (NEI-VFQ). Previous publications (22) report extensively on this first phase, including detailed methods and recruitment summary and concordance of the telemedicine eye screening findings and comprehensive examination diagnosis (23).

All participants who completed Visit 2 or who were fast-tracked were invited to participate in Phase 2 of the study. Phase 2 was a randomized controlled clinical trial designed to evaluate whether an enhanced intervention (EI) using patient navigation and social worker support improved patient adherence to follow-up eye care over usual care (UC) among those with newly diagnosed or suspected glaucoma or other ocular conditions.

**Recruitment and randomization**. Participants consenting to participate in Phase 2 were randomized to either the UC group or the EI group at a fixed 1:1 allocation ratio by using a masked method of random permuted blocks. Study coordinators retrieved the randomization and allowed participants to select an ophthalmologist they would like to follow up with over the next several years from a list of 20 participating offices located within 5 miles of the screening site.

**Usual care**. Participants randomized to UC were given their selected ophthalmologist’s contact information and a copy of their eye examination results. UC participants were instructed to schedule an initial appointment with the ophthalmologist (Visit 3). Once connected to that ophthalmologist, services provided by each local ophthalmology practice generally included telephone calls and/or text message reminders before appointments. No practices provided patient navigator or social worker assistance as part of their usual care during the study period.

**Enhanced intervention**. Participants randomized to EI received a team-based intervention that included comprehensive assessment by a licensed social worker and assistance from patient navigators. The social worker called EI participants up to 3 times within 2 weeks to conduct an initial assessment, explain the EI process, assess participants’ understanding of their new or existing ocular diagnosis, and document current and past barriers to obtaining eye care. The social worker provided community resources for participants in need of food and medications at no cost or at a reduced cost and discussed options for transportation to the local ophthalmologist. The social worker also assessed the participant’s ability to complete their activities of daily living and provided emotional support. EI participants interacted by telephone with the social worker at least 3 times per year over 2 years.

Wills Eye Hospital study managers, ocular technicians, and research assistants served as patient navigators for EI participants. Their responsibilities included calling participants to schedule appointments; confirming appointments by mail, email, and/or text messaging; arranging transportation through Customized Community Transportation and Philadelphia Paratransit Service; and scheduling language interpreters with medical training to participate in eye examinations as needed. Patient navigators were able to identify cultural and language differences and were aware of health literacy issues. When possible, navigators were race and language concordant with the patient population.

**Management and follow-up examinations**. At Visit 3 and each follow-up visit, the local ophthalmologist assessed the participant’s ocular, medical, and family history and conducted a comprehensive eye examination based on their clinical practice. The ophthalmologist would reconfirm ocular diagnoses, perform testing, adjust treatment recommendations as needed, and recommend follow-up intervals for the participant.

**Final study visit**. All randomized participants were invited to a final visit at their PCP’s office at the end of the follow-up period. At this visit, the NEI-VFQ was re-administered, visual acuity and IOP were measured, and overall participant satisfaction with the study was assessed.

**Outcome assessment**. The research staff visited local ophthalmologists’ offices to record visit dates, indications, findings, and treatments for up to 3 years from Visit 2. Data collection closed in March 2019.

**Annual adherence**. The primary outcome measure was adherence to recommended follow-up eye care appointments after Visit 3. Adherence was assessed annually on the basis of the expected follow-up schedule defined at the index visit for that year. In the first year, the follow-up recommendation given at Visit 3 by the ophthalmologist was classified into 1 of 4 categories: return within 2 months, return in 3 to 4 months, return in 6 months, or return in 12 months. This follow-up recommendation was then translated into the corresponding expected number of visits per year: 6, 3, 2, or 1. Participants were classified as adherent if the number of visits made within 13 months of Visit 3 (395 days) met or exceeded this expected number. Those who attended fewer visits or did not attend the initial visit with the local ophthalmologist within 12 months were deemed nonadherent for the first year. Adherence in the second and third years of follow-up was similarly defined; however, the follow-up recommendation used to define the required number of visits was based on the patient’s most recent visit with the ophthalmologist before the start of that follow-up year. That is, the last visit that occurred during the first year of follow-up determined the follow-up schedule applied to the second year; similarly, the last visit that occurred during the second year of follow-up determined the follow-up schedule for the third year. When no visit occurred during a given year, the previous follow-up recommendation was carried forward. Additional measures of intervention effectiveness were explored, as detailed below.

**Visit 3 attendance — initial visit with local ophthalmologist**. The study evaluated the intervention’s effectiveness in achieving the initial connection with the local ophthalmologist through timely attendance at Visit 3. Timely attendance was defined as having a first visit within 12 months of randomization.

**Visit 4 attendance — first follow-up visit with local ophthalmologist**. Adherence to the first follow-up visit (Visit 4) was assessed on the basis of the follow-up recommendation of the local ophthalmologist at Visit 3. Participants with follow-up recommended within 2 months were deemed adherent to the first follow-up visit if they returned within 3 months; for recommended follow-up of 3 to 4 months, 6 months, or 12 months, patients were considered adherent to Visit 4 within 6, 12, or 15 months, respectively.

**Total number of visits with local ophthalmologist**. The total number of visits included all visits on distinct days occurring after randomization, including Visit 3.

**Satisfaction**. A brief questionnaire was administered at the final study visit to assess overall satisfaction. By using a 4-point Likert-type scale, participants were asked to state their satisfaction with the study and the local ophthalmologist, perceived helpfulness of the study toward understanding their recommended eye-care, and likeliness to continue with follow-up care at the local ophthalmologist.

### Statistical analysis

The study was designed to detect a 20% difference in adherence rates between groups during the first year of follow-up by using a 2-tailed test with α = 0.025. With a final sample size of at least 135 participants per group, power to detect such a difference was 86% when the overall adherence across both study arms was 50%.

Participant characteristics at Visits 1 and 2 were summarized by randomization arm by using means and SDs or number and percentages. Adjusted estimates of the relative risk (aRR) of timely attendance at Visit 3 were calculated by using Poisson regression in a generalized estimating equation framework (24). An extension of this model for longitudinal data was used to jointly model repeated annual measures of follow-up adherence (25). The longitudinal model included time (year 1, 2, or 3), randomization assignment, and randomization by time interaction. Both models adjusted for baseline characteristics believed to be associated with adherence to follow-up: Visit 2 recommended follow-up (as a surrogate for disease severity), age at screening, sex, insurance type, and baseline NEI-VFQ composite score.

In the longitudinal model, 2 relative risks were calculated and 2 hypotheses were tested: 1) comparing randomization groups at year 1 to assess differences in early adherence and 2) calculating the average effect of randomization group across years 2 and 3 to test the long-term efficacy of the intervention. Each test was performed with α = 0.025. Supporting analyses compared groups with respect to the percentage of participants who attended any visits with the local ophthalmologist, the percentage of participants who were adherent to their first post-Visit 3 visit, and the total number of visits after randomization. Analyses of dichotomous end points used the same approach as Visit 3 analysis. Number of visits was modeled by Poisson regression with follow-up time from randomization as the offset. An exploratory subset analysis was performed for participants with glaucoma-related diagnoses (those diagnosed with glaucoma, glaucoma suspect, or ocular hypertension). All analyses were performed by using SAS 9.4 and SAS/STAT 14.3 (SAS Institute).

## Results

### Participant characteristics

**Figure 1:**
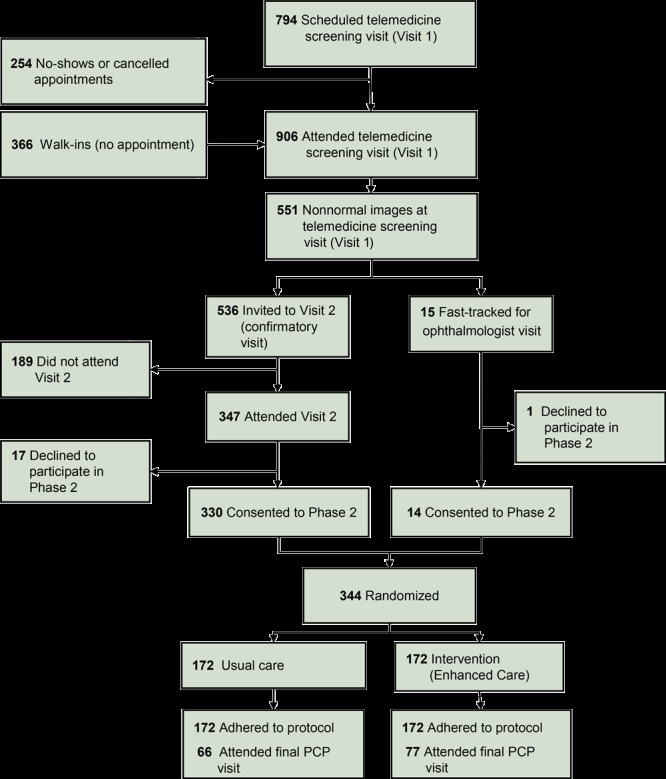
Flow chart describing the Philadelphia Telemedicine Glaucoma Detection and Follow-up Study, indicating participant inclusion, exclusion, and randomization to the usual care group or enhanced intervention group.

From April 2015 through February 2017, 906 participants completed the telemedicine eye vision screening (Visit 1) with their PCP as part of Phase 1 of this 5-year study (Figure 1) (22). On telemedicine reading, 355 participants (39%) were classified as having normal fundus images. The remaining 551 participants had abnormal or suspicious fundus images (334, 37%), unreadable fundus images (155, 17%), or IOP exceeding 21 mm Hg (62, 7%). Fifteen participants had IOP >30 mm Hg that required fast-track referral to the local ophthalmologist. The other 536 participants were invited to have a comprehensive eye examination by their PCP; 347 participants (65%) attended this Visit 2. These 347 patients and the 15 fast-tracked patients were invited to participate in Phase 2. A total of 344 participants consented and were randomized to either EU (n = 172) or UC (n = 172). Participants were followed up for a minimum of 22 months post-randomization.

The mean age of participants was 59.9 years at screening; most (59%) were women and 66% were African American (Table 1). Roughly two-thirds (n = 230, 66.9%) had a glaucoma-related diagnosis at Visit 2 or were fast-tracked to visit an ophthalmologist because of high IOP. An NEI-VFQ average composite score of 82 indicated somewhat diminished vision-related quality of life. We saw no large differences in randomization groups with respect to baseline characteristics, although the EI group had a slightly higher percentage of women and a lower percentage of participants with diabetes (Table 1).

**Table 1:** Demographic and Clinical Characteristics of Subjects (N = 344) Randomized to Usual Care and Intervention Groups, the Philadelphia Telemedicine Glaucoma Detection and Follow-up Study

Characteristic	All (N = 344)	Usual Care (n = 172)	Intervention (n = 172)
**Age, y, mean (SD)**	59.9 (11.0)	59.0 (10.6)	60.8 (11.4)
**Sex, n (%)**			
Female	202 (58.7)	94 (54.7)	108 (62.8)
Male	142 (41.3)	78 (45.3)	64 (37.2)
**Race/ethnicity^a^, n (%)**			
African American	223 (66.2)	111 (66.1)	112 (66.3)
White	52 (15.4)	25 (14.9)	27 (16.0)
Asian	16 (4.8)	7 (4.2)	9 (5.3)
Hispanic	37 (11.0)	20 (11.9)	17 (10.1)
More than one race	9 (2.7)	5 (3.0)	4 (2.4)
**Family of history glaucoma, n (%)**	87 (25.3)	49 (28.5)	38 (22.1)
**Current smoker, n (%)**	95 (27.6)	45 (26.2)	50 (29.1)
**Hypertension, n (%)**	237 (68.9)	122 (70.9)	115 (66.9)
**Diabetes, n (%)**	198 (57.6)	108 (62.8)	90 (52.3)
**Insurance type, n (%)**			
Medicaid	130 (37.8)	64 (37.2)	66 (38.4)
Medicare	91 (26.5)	40 (23.3)	51 (29.7)
Private	99 (28.8)	54 (31.4)	45 (26.2)
None	24 (7.0)	14 (8.1)	10 (5.8)
**Screening outcome, n (%)**			
Abnormal	218 (63.4)	112 (65.1)	106 (61.6)
Unreadable	85 (24.7)	38 (22.1)	47 (27.3)
Ocular hypertension	41 (11.9)	22 (12.8)	19 (11.0)
**Visit 2 recommended follow-up, n (%)**			
Every 3–4 months	54 (15.7)	26 (15.1)	28 (16.3)
Every 6 months	115 (33.4)	59 (34.3)	56 (32.6)
Every 12 months	175 (50.9)	87 (50.6)	88 (51.2)
**logMAR visual, mean (SD)**			
Lower (better)	0.2 (0.2)	0.2 (0.2)	0.2 (0.2)
Higher (worse)	0.3 (0.4)	0.3 (0.5)	0.3 (0.3)
**IOP^b^, mmHg, mean (SD)**			
Lower (better)	14.9 (4.4)	15.3 (4.7)	14.5 (4.0)
Higher (worse)	16.6 (5.2)	16.9 (5.3)	16.2 (5.0)
**C/D Ratio^c^, mean (SD)**			
Lower	0.4 (0.2)	0.4 (0.2)	0.4 (0.2)
Higher	0.5 (0.2)	0.5 (0.2)	0.5 (0.2)
**Mean deviation^d^, dB, mean (SD)**			
Lower	4.7 (5.2)	4.8 (5.5)	4.7 (4.9)
Higher	7.8 (6.3)	8.0 (6.6)	7.5 (5.9)
**Glaucoma-related diagnosis, n (%)**			
None	114 (33.1)	52 (30.2)	62 (36.0)
Glaucoma	38 (11.0)	17 (9.9)	21 (12.2)
Glaucoma suspect	153 (44.5)	80 (46.5)	73 (42.4)
Ocular hypertension	25 (7.3)	14 (8.1)	11 (6.4)
Fast-tracked at screening (IOP >30 mm Hg)	14 (4.1)	9 (5.2)	5 (2.9)
**NEI-VFQ composite score^e^, mean (SD)**	82.2 (15.7)	82.1 (16.0)	82.3 (15.5)

Abbreviations: C/D ratio, cup-to-disc ratio; dB, decibel; IOP, intraocular pressure; logMAR, logarithm of the minimum angle of resolution; NEI-VFQ, National Eye Institute Visual Function Questionnaire.

a Race was unknown for 7 subjects.

b IOP was carried forward from visit 1 for 17 subjects (including 14 fast-tracked subjects).

c C/D ratio was not available for 22 subjects (including 14 fast-tracked subjects).

d Mean deviation was not available for 19 subjects (including 14 fast-tracked subjects).

e One subject did not complete the questionnaire; samples sizes vary across subscales.

**Timely Visit 3 attendance**. About half of participants (56.7% [74.4% EI, 39.0% UC]) attended the initial visit with the local ophthalmologist within 12 months of randomization (Table 2). In adjusted analysis (Table 2), the EI group showed an 85% relative increase in timely Visit 3 attendance (adjusted relative risk [aRR] = 1.85; 95% CI, 1.51–2.28; *P* < .001). The effect was similar in the subset of participants with glaucoma-related diagnoses (aRR = 1.73; 95% CI, 1.37–2.19; *P* < .001). Among those who made timely contact with the local ophthalmologist, the median time to first visit was 57 days (interquartile range [IQR]: 39–92) in EI and 47 days (IQR: 27–82) in UC. Rates of any attendance at the local ophthalmologist were also higher in EI (77.9% vs 41.3%; aRR = 1.83; 95% CI, 1.51–2.22; Table 2) although only 6 EI and 4 UC participants ever attended a later visit after failing to make contact with the ophthalmologist in the first 12 months.

**Table 2:** Summary of Adherence Outcomes, the Philadelphia Telemedicine Glaucoma Detection and Follow-up Study

Outcome	Usual Care (n = 172) n (%)	Intervention (n = 172) n (%)	All (n = 344) RR (95% CI)	Glaucoma (n = 230) RR (95% CI)
Attended Visit 3 within 12 months	67 (39.0)	128 (74.4)	1.85 (1.51–2.28)	1.73 (1.37–2.19)
Attended any visit at local ophthalmologist	71 (41.3)	134 (77.9)	1.83 (1.51–2.22)	1.69 (1.36–2.09)
Adherent in Year 1	14 (8.1)	32 (18.6)	2.08 (1.14–3.76)	2.30 (1.10–4.82)
Adherent to first follow-up visit (Visit 4)	39 (22.7)	97 (56.4)	2.39 (1.78–3.22)	2.55 (1.79–3.63)
At least 1 visit in Year 1	31 (18.0)	71 (41.3)	2.18 (1.52–3.12)	2.31 (1.48–3.62)
Total visits attended per year	0.4 (0.7)^a^	0.9 (0.8)^a^	2.07 (1.54–2.78)	1.99 (1.44–2.74)

Abbreviation: RR, relative risk.

a Values are mean (SD).

**Figure 2:**
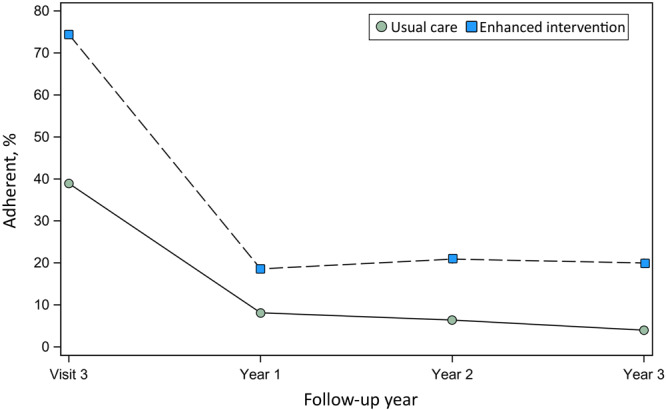
Adherence to recommended follow-up schedule over time by intervention group. Visit 3 was the initial visit with the community ophthalmologist. Timely adherence to Visit 3 was defined as attendance within 12 months of randomization. Annual adherence in Years 1–3 was defined as having attended all recommended follow-up visits within 13 months based on the recommended follow-up at the visit closest to the beginning of the year.

**Adherence to follow-up after Visit 3**. In year 1, the adherence rate was 18.6% in the EI group and 8.1% in UC with an aRR of 2.08 (95% CI, 1.14–3.76; *P* = .016) indicating that the intervention significantly increased the rate of adherence (Figure 2) (Table 3). Adherence was relatively stable in years 2 and 3 for EI, while declining over time in UC. The average aRR of adherence across years 2 and 3 was 3.92 (95% CI, 1.24–12.43; *P* = .02). Results were similar in an exploratory analysis of the glaucoma-related diagnosis subset with an aRR of 2.30 for year 1 adherence (95% CI, 1.10–4.82) and 3.44 across years 2 and 3 (95% CI, 1.11–10.63) (Table 3).

**Table 3:** Intervention Effect on Adherence to Follow-up Schedule, by Year of Follow-up, the Philadelphia Telemedicine Glaucoma Detection and Follow-up Study

Variable	Total	Adherent to Follow-Up, n (%)	Adjusted Relative Risk (95% CI)	*P* Value^a^
**Year 1**				
Usual care	172	14 (8.1)	Reference	NA
Intervention	172	32 (18.6)	2.08 (1.14–3.76)	.02
**Year 2**				
Usual care	140	9 (6.4)	Reference	NA
Intervention	129	27 (20.9)	2.90 (1.39–6.02)	.004
**Year 3**				
Usual care	25	1 (4.0)	Reference	NA
Intervention	25	5 (20.0)	5.30 (0.56–49.95)	.15
**Average, year 1–year 2**				
Usual care	NA	NA	Reference	NA
Intervention	NA	NA	3.92 (1.24–12.43)	.02
**Glaucoma Subset**				
**Year 1**				
Usual care	120	9 (7.5)	Reference	NA
Intervention	110	21 (19.1)	2.30 (1.10–4.82)	.03
**Year 2**				
Usual care	98	7 (7.1)	Reference	NA
Intervention	79	16 (20.3)	2.47 (1.05–5.80)	.04
**Year 3**				
Usual care	19	1 (5.3)	Reference	NA
Intervention	18	4 (22.2)	4.80 (0.56–41.17)	.15
**Average, year 2–year 3**				
Usual care	NA	NA	Reference	NA
Intervention	NA	NA	3.44 (1.11–10.63)	.03

Abbreviation: NA, not applicable.

a *P* values were calculated by using GEE (generalized estimating equation) Poisson regression models adjusted for Visit 2 recommended follow-up (as a surrogate of disease severity), age at screening, sex, insurance type, and baseline National Eye Institute Visual Function Questionnaire composite score.

For adherence to the first follow-up visit recommended by the local ophthalmologist (Visit 4), the rate was 56.4% for EI group and 22.7% for UC (aRR = 2.39; 95% CI, 1.78–3.22) (Table 2). The average number of visits per year of follow-up was 0.9 in EI and 0.4 in UC (aRR = 2.07; 95% CI, 1.54–2.78) (Table 2). The proportion of participants who attended at least 1 visit in the first year of follow-up was 41.3% in EI and 18.0% in UC (aRR = 2.18; 95% CI, 1.52–3.12) (Table 2).

**Final study visit and satisfaction survey**. One-hundred forty-three participants attended the final study visit at the PCP office (EI, 77; UC, 66). Both groups were satisfied or very satisfied with participation in the study (EI, 98.7%; UC, 93.9%), and most participants in both groups found the study very helpful in understanding and taking care of their eyes (EI, 64.9%; UC, 54.5%).

## Discussion

In analysis of our primary outcome, we found that an intervention combining the support of patient navigators and social workers doubled adherence to recommendations for follow-up with a local ophthalmologist during the first year. These effects were similar for participants with or without glaucoma-related diagnoses. Much of this effect was likely due to an 85% relative increase in timely attendance at the initial visit with the ophthalmologist (Visit 3) for those randomized to the intervention arm (EI). After the first year, adherence rates dropped, but were still higher in the EI group.

Our results are similar to previous studies. The UC group in our study had only 39% attendance at the initial ophthalmologist visit, similar to results from the Hoffberger program, which provided free community-based eye screenings to residents of Baltimore, Maryland, at high risk for eye disease (6). In another prospective study, after 1 year, participants with glaucoma-related diagnoses had 82.5% follow-up adherence rates with the help of only patient navigators in office-based settings, compared with 73.3% in the usual care group; however, differences were not significant (12). Adherence in this study was defined as 1 or more visits within 1 year of diagnosis, and these rates were similar to what we observed for the same outcome in our intervention group (74.4%). Although the UC rate was much lower in our study, it is consistent with low rates seen in another recent study (26).

Patients with glaucoma may face barriers to receiving follow-up eye care, which should be recognized and addressed. A questionnaire presented to patients in a glaucoma clinic who were referred to a medical social worker found that the most frequent barrier to receiving eye care was emotional distress; additional barriers were cost of visits, lack of insurance, transportation, impairment of daily activities, and language (7). Another study reported forgetfulness as a major barrier to adherence to follow-up care (27). Degree of depression was also correlated with level of nonadherence to eye care recommendations (28). The results of our study suggest that combining support of patient navigators and social workers may be effective in reducing these barriers and thereby improving outcomes.

Our low annual adherence rates may be because adherence for the year was defined on the basis of the follow-up recommendation at the beginning of the year. For example, if a participant was given a recommendation during Visit 3 to follow up in 2 months, this was considered the desired follow-up interval throughout the following year. However, recommendations for follow-up could have varied during the year, and this may have affected our annual adherence results.

Our study had several limitations. First, in spite of the improved adherence in the EI, annual adherence was still unacceptably low compared to what is necessary for adequate treatment. Second, our sample size for year 3 limited our ability to assess the long-term benefit of the intervention. Lastly, different ophthalmologists’ offices used diverse measures to remind patients to return for follow-up eye examinations, which were not controlled and could have affected our results.

This study targeted a diverse, urban population at risk for glaucoma but not receiving regular eye care. Study results would likely be generalizable to similar settings, although access to care, insurance rates, and existing support systems are likely to differ in other geographic areas and may affect the benefit of the intervention.

Future studies could consider combining additional interventions to further increase rates of adherence to follow-up eye care. Other interventions that may show promise include providing incentives such as free eyeglass prescriptions or free eyeglasses (26) and providing other financial incentives to encourage at-risk participants to return for follow-up eye examinations (12). The costs of the screening phase have been previously reported (22); the cost-effectiveness of our adherence intervention is being evaluated. In conclusion, our study addresses a critical gap in ophthalmic care by improving adherence to follow-up recommendations by using patient navigators and social workers. Addressing this gap is important because adherence to eye care contributes to a better prognosis for patients with chronic eye disease. We believe that use of social workers and patient navigators could be scaled on a national level to decrease the growing burden associated with glaucoma and other sight-threatening eye diseases.
